# Effectiveness of seminar-case learning for use in practice teaching of statistics for undergraduates majoring in preventive medicine: a prospective cluster-randomized controlled trial

**DOI:** 10.1186/s12909-022-03297-8

**Published:** 2022-04-02

**Authors:** Wei-wei Chang, Li-jun Zhu, Li-ying Wen, Jian-gen Song, Yun-fei Zou, Yue-long Jin

**Affiliations:** Department of Epidemiology and Health Statistics, School of Public Health, Wannan Medical College, Wuhu, 241002 Anhui China

**Keywords:** Seminar-case learning, Students, Statistics

## Abstract

**Background:**

The seminar-case learning (SCL) method is a case-oriented teaching model, with teachers and students as the main body of teaching, characterized by communication, interaction, and mutual inspiration. This study explored the effects of the SCL method versus traditional lecture-based learning (LBL) in the statistics curriculum for undergraduate students majoring in preventive medicine. Research questions were: 1) whether the scores of students in the experimental group (the SCL model) were higher than those in the control group (the LBL model); 2) whether the students’ satisfaction in the experimental group was better than that in the control group; and 3) whether the self-report benefit of students in the experimental group was better than that in the control group.

**Methods:**

We conducted a two-armed cluster-randomized education intervention trial in practice teaching of health statistics among undergraduates majoring in preventive medicine. Two administrative classes (classes 1–4 and classes 5–8) were divided into the experimental group and the control group according to the principle of drawing lots. The students in two groups received the same statistical theory course. For the arrangement of statistical practice course, the experimental group adopted the SCL model, and the control group used the LBL model. The teaching effect was evaluated via an examination and an anonymous questionnaire survey.

**Results:**

Scores for noun explanation questions in the experimental group showed no statistical significance with that of the control group(*U* = 2911.0, *P* = 0.964). The scores of single choice, calculation, and case analysis questions, and the total scores were significantly higher than that of the control group (*P* < 0.05). Students’ satisfaction with arrangements of the practice course in the experimental group (92.41%) was significantly higher than that of in the control group (77.03%), the difference was statistically significant (*χ*^*2*^ = 7.074, *P* = 0.008). The self-report benefit of students in the experimental group was better than that in the control group (*P* < 0.05).

**Conclusion:**

As an effective method of high-quality education, the SCL model is worthy of further promotion in the practice teaching of preventive medicine.

## Background

Chinese higher education has long focused on knowledge teaching and ignores the cultivation of students’ thinking ability. Traditional lecture-based learning (LBL) is mainly based on the teacher. Teachers instill knowledge from books to students through a teacher-centered teaching model, which fails to cultivate ability of independent thinking, autonomous learning, and problem solving [1]. With the transformation of medical and public health service models, society has higher requirements for the technical level and the ability of solving practical problems of public health talents [2]. However, some problems remain in China, such as insufficient professional quality of such talents and imperfect education model, which cannot meet the challenges of public health emergencies and meet the requirements of heavy public health tasks [3]. How to cultivate high-quality application-oriented talents to meet the growing needs of public health in China has therefore become an important issue.

Health statistics is a science dealing with the collection, analysis, interpretation, and presentation of numerical data in the field of health services, which is a mainstream curriculum in the field of preventive medicine and public health recognized at home and abroad [4]. It is a compulsory professional curriculum for preventive medicine students and its teaching purpose is to cultivate students’ logical thinking and problem-solving abilities [5]. Although statistics is very important in the training of medical students, those in various medical schools generally consider statistics difficult to learn, difficult to understand, and even unable to apply what they have learned [4, 6–8]. Practice teaching of health statistics is to further consolidate students’ grasp of theoretical knowledge. The traditional practical teaching mode is mainly teacher-centered. The teacher passes the teaching contents to the students by explaining cases, and the students passively accept the knowledge and finish the homework exercises. Although the traditional mode can meet the teaching needs of undergraduates majoring in preventive medicine, it is somewhat lacking in terms of stimulating students’ learning initiative, and lack of active thinking ability in practical work, which is difficult to meet the needs of contemporary society for public health and preventive medicine talents [3].

Originating from the University of Berlin in Germany, the seminar teaching method is a teaching model in which students work in small groups to discuss a particular issue under the guidance of teachers [9]. Students take the initiative to learn the course content assigned by the teacher in advance, find the answers to the questions assigned before the course, and share the knowledge points with other students in the course [10]. This teaching method focuses on the interaction between students and teachers in class, which can fully mobilize students’ subjective initiative and cultivate their divergent thinking and innovative thinking [11]. The method has been applied in many medical subjects and may lead to an increase in knowledge [12–15]. In case-based learning (CBL), teachers provide real cases, which include sufficient information and details to induce students’ active analysis and stimulate interest in learning. CBL can improve students’ logical thinking and ability to analyze and solve problems [16].

Seminar and CBL teaching methods are effective in medical courses [12–15, 17–19], but the combination of them (seminar-case learning, SCL) has not been reported in literature. We hypothesize that,compared with the LBL model, the SCL model can improve the teaching effect of statistical practice. Therefore, we conducted a prospective randomized controlled trial in practice teaching of health statistics among undergraduates majoring in preventive medicine. The purposes was to assess the following: (1) Whether the scores of students in the experimental group (the SCL model) were higher than those in the control group (the LBL model). (2) Whether the students’ satisfaction in the experimental group was better than that in the control group. (3) Whether the self-report benefit of students in the experimental group was better than that in the control group.

## Methods

### Participation and design

The study participants were from third-grade undergraduates majoring in preventive medicine, School of Public Health, Wannan Medical College (Anhui province in China) (fall semester of 2021). There are five grades of undergraduates majoring in preventive medicine, with 150–200 students in each grade. Each grade has eight classes with 20–30 students in each class. Health statistics is a compulsory curriculum for students majoring in preventive medicine, which is arranged in third-grade.

This was a prospective, two-centre, open-labelled, cluster-randomized education intervention trial lasting 4 months with clusters defined as administrative classes within the settlements. There are a total of eight classes for third-grade. According to the arrangement of the Academic Affairs Office of the school, the eight classes are divided into two administrative classes (classes 1–4 and classes 5–8), with 116 students in classes 1–4 and 105 students in classes 5–8, respectively. After careful consideration and deployment by the director of the Department of Epidemiology and Health Statistics, two teachers with the same professional title (both associate professors) and the same teaching experience in statistics (≥ 10 years) were selected to participate in the teaching of statistics and one teacher was selected as the teacher of the experimental group. We put two class numbers (1–4 and 5–8) into an opaque box, and the teacher of the experimental group drew a number from it as the experimental group. Finally, classes 1–4 were determined as the experimental group and classes 5–8 as the control group.

All the students in the two administrative classes participated in the theoretical and experimental courses of health statistics. After learning, students voluntarily participated in the questionnaire survey. A total of 221 questionnaires were distributed to students and 176 questionnaires were recovered, of which 23 were unqualified. The 23 invalid questionnaires included: (1) 15 questionnaires with too many missing answers (accounting for or exceeding 15% of the total numbers of questions); (2) 5 questionnaires with the same or most of the same options for each question; (3) 3 questionnaires with wrong options without following the instructions (For example, there are two choices in the single choice question).Finally, there were 153 valid questionnaires, of which classes 1–4 and 5–8 were 79 and 74, respectively.Written informed consent forms were obtained from the subjects that participated in this study.

### Intervention contents

Statistics is a curriculum including 108 class hours (theory 72, practice 36), and the textbook used is health statistics (eighth edition, edited by Li Xiaosong, People's Medical Publishing House) [20]. The main practice teaching contents included descriptive statistics, binomial distribution and normal distribution, *t*-test, analysis of variance, Chi-square (χ^2^) test, nonparametric statistics, correlation and regression, statistical chart, and multivariate statistical analysis.

To ensure the consistency of theoretical teaching between the two groups as far as possible, with the assistance of the director of the teaching and research office and all the members, the two teachers prepared and taught lessons uniformly according to the knowledge points (including key points and difficulties) of the teaching contents involved in the syllabus and adopted the same theory teaching model (PPT + blackboard deduction) in the classroom. The theoretical teaching hours and contents of the two groups were completely consistent. For the arrangement of statistical practice course, the experimental group adopted the SCL model, and the control group used the LBL model. Both groups were taught in the classroom for practical teaching.

The SCL model was as follows: (1) Preparation work (1 week). Based on the course content, the teacher assigned seminar teaching tasks (statistics cases) in advance, set aside heuristic questions, and briefly introduced and arranged the topics, personnel, and progress of the discussion. In small groups, students needed to collect the relevant literature, conducted independent discussions, and prepared answers to questions, (2) Classroom explanation (40 min): Each group selected one main presenter to report the documents and problem-solving ideas that they had compiled, and the rest of the group could provide relevant supplements, (3) Questions and debates (20 min): Regarding the statistical cases, the whole class could communicate, ask questions, give opinions, and explain and supplement the content of the report. At the same time, teachers should guide the discussion in cases of disagreements among students, (4) Summary (20 min) in classroom: The teacher briefly evaluated the speaker’s speech, the performance of the group members, and the results of the class discussion, summarized the important and difficult knowledge of this lesson, and clearly highlighted the advantages, problems, and needs of improvement in seminar teaching in this lesson, and deployed the tasks and arrangements for the next seminar. The whole teaching time of each lesson was 80 min once a week.

The traditional LBL model was as follows: the teacher arranged exercises in class (the same as the experimental group). The cases used by both groups of students are from the Statistics Experiment Instruction Manual. The teacher made a simple analysis of the problem-solving ideas of the exercises. After class, the teacher summarized and evaluated the students’ homework on the practical exercises. The whole teaching time of each lesson was 80 min once a week.

### Evaluation of teaching effects

After 16 weeks of theoretical and practical learning, the two groups underwent the same examinations and an anonymous questionnaire survey, including: (1) A theoretical examination (a total score of 100 points): including single choice questions (50 points), noun explanation questions (14 points), calculation questions (28 points), and case analysis questions (8 points). Noun explanation questions are used to test students’ understanding and mastery of basic theoretical knowledge of statistics. Single choice questions, calculation questions, and case analysis questions are used to assess students’ comprehensive statistical application ability. (2) The questionnaire, included (i) basic information: gender (male, female), age (year), math scores of the college entrance examination(< 90, 90–120, 120–150), and work experience in scientific research(yes, no). (ii) Students’ satisfaction included three items: arrangements of theory courses, arrangements of practice courses, and the teaching effect of teachers. Satisfaction is divided into five levels: 1 = very satisfied, 2 = satisfied, 3 = general, 4 = dissatisfied, 5 = very dissatisfied. The Cronbach’s α was 0.847, and the Kaiser–Meyer–Olkin (KMO) value was 0.723 (*P* < 0.05).The number of students who were satisfied with the teaching effect was the sum of those who were very satisfied, and satisfied. (iii) Students’ perceptions of self-reported benefits of studying statistics included four items: help future work and study (a lot, some, no), improve scientific research thinking ability (a lot, some, no), helpful for scientific research (a lot, some, no), and improve statistical application capability (a lot, some, no). The Cronbach’s α was 0.745, and the KMO value was 0.679 (*P* < 0.05).

### Data analysis

Data were analyzed using SPSS 25.0 software. For qualitative variables, the rates or constituent ratios were used for descriptive statistics.The satisfaction between the two groups was compared by χ^2^ test. The Kolmogorov–Smirnov (K-S) test was used to assess the normality of examinations scores. The results of K-S test concluded that the scores did not accord with normality (*P* < 0.05) and scores were expressed as medians M (*P*_*25*_, *P*_*75*_) for descriptive analysis. The scores of theoretical examination and student self-reported benefit rate between the two groups were compared by using the Mann–Whitney U test. A value of *P* < 0.05 (two-tailed) was considered statistically significant.

## Results

All 153 students underwent the same examinations and an anonymous questionnaire survey. There were 79 students in the experimental group, including 40 males (50.63%). A total of 74 students were in the control group, including 32 males (43.24%). There were no statistically significant differences in gender, math scores of college entrance examination and scientific research work experience between the two groups (*P* > 0.05, Table 1).

**Table 1:** Comparison of general data between two groups of students [n(%)] (*n* = 153)

Variables	Group	Experimental group (*n*=79)	Control group (*n*=74)	*χ*^*2*^	P*
Gender	Male (*n*=72)	40(50.63)	32(43.24)	0.837	0.360
	Female (*n*=81)	39(49.37)	42(56.76)		
Math scores of college entrance examination	<90 (*n*=11)	5(6.33)	6(8.11)	0.634	0.728
	90-(*n*=116)	62(78.48)	54(72.97)		
	120-150 (*n*=26)	12(15.19)	14(18.92)		
Scientific research work experience	Yes (*n*=34)	17(21.52)	17(22.97)	0.047	0.848
	No (*n*=119)	62(78.48)	57(77.03)		

^*^Chi-square (χ^2^) test

The scores of theoretical examinations were shown in Table 2. Scores for noun explanation questions in the experimental group showed no statistical significance with that of the control group (*U* = 2911.0, *P* = 0.964). The scores of single choice, calculation, and case analysis questions, and the total scores were significantly higher than that of the control group (*P* < 0.05, Table 2).

**Table 2:** Comparison of examinations scores between the two groups [ M (*P*_*25*_*, P*_*75*_)]

Scores of different test Questions	Experimental group (*n* = 79)	Control group (*n* = 74)	*U*	P*
Single choice questions	41 (35,44)	37 (32,40)	1746.0	< 0.001
Noun explanation questions	14 (12,16)	13 (11,16)	2911.0	0.964
Calculation questions	26 (23,26)	22 (19, 24)	1417.5	< 0.001
Case analysis questions	6 (3, 7)	3 (2, 4)	1344.0	< 0.001
Total	83 (76, 91)	73 (67, 81)	1551.5	< 0.001

^*^the Mann–Whitney U test

Students’ satisfaction with arrangements of the practice course in the experimental group (92.41%) was significantly higher than that of in the control group (77.03%), the difference was statistically significant (*χ*^*2*^ = 7.074, *P* = 0.008). There was no statistically significant difference in satisfaction with the arrangement of theoretical courses and teaching effect of teachers between the two groups (*P* > 0.05, Table 3).

**Table 3:** Comparison of students' satisfaction between the two groups [n(%)](*n* = 153)

Satisfaction	Experimental group (*n* = 79)	Control group (*n* = 74)	*χ*^*2*^	P*
The arrangement of theory courses	69(87.34)	62(83.78)	0.393	0.531
The arrangement of practice courses	73(92.41)	57(77.03)	7.074	0.008
The teaching effect of teachers	72(91.14)	61(82.43)	2.549	0.110

^*^Chi-square (χ^2^) test

There were significant improvements in scientific research thinking ability and statistical application capability in the experimental group compared to the control group (*P* < 0.05). Nearly half of the students reported that statistics would be of great help to future work, study, and scientific research, and this proportion was much higher than the control group (*P* < 0.05, Table 4).

**Table 4:** Comparison of self-reported benefits between two groups [n(%)] (*n* = 153)

Questions	Group	Experimental Group (*n* = 79)	Control group (*n* = 74)	*U*	P*
Help future work and study	A lot (*n* = 62)	41(51.90)	21(28.38)	2216.5	0.003
	Some (*n* = 90)	38(48.10)	52(70.27)		
	No (*n* = 1)	0(0.00)	1(1.35)		
Improve scientific research thinking ability	A lot(*n* = 36)	25(31.65)	11(14.86)	2333.5	0.006
	Some (*n* = 109)	52(65.82)	57(77.03)		
	No(*n* = 8)	2(2.53)	6(8.11)		
Helpful for scientific research	A lot (*n* = 58)	40(50.63)	18(24.32)	2218.5	0.003
	Some (*n* = 91)	36(45.57)	55(74.33)		
	No (*n* = 4)	3(3.80)	1(1.35)		
Improve statistical application capability	A lot (*n* = 55)	38(48.10)	17(22.97)	2184.0	0.002
	Some (*n* = 93)	39(49.37)	54(72.97)		
	No(*n* = 5)	2(2.53)	3(4.06)		

^*^the Mann–Whitney U test

## Discussion

Health statistics, is an important professional course in field of preventive medicine education. Students are required to master the basic theory and methods of medical statistics, and the course focuses on cultivating students’ scientific thinking and inference ability, which can lay a good statistical foundation for future scientific research [4]. Even if students have a high interest in learning statistics and a good grasp of the contents that teachers are teaching, they have difficulty in applying health statistics methods to scientific research or health services [21]. Traditional teaching is teacher-centered classroom learning, which emphasizes the delivery of syllabus and concepts [22]. Students learn passively, which leads them to not solve practical problems when they encounter them [23]. Statistics teaching is a teacher’s restatement of the theoretical content of textbooks, which ignores the cultivation of students’ thinking ability. Students have low levels of enthusiasm for boring classroom learning [24]. Although the traditional practice teaching model can meet the teaching needs of preventive medicine students, it is deficient in terms of students’ learning initiative and lacking active thinking ability in practice.

In our study, the satisfaction with arrangements of practice course, the scores of single choice, calculation, and case analysis questions, and the total scores were significantly higher in the SCL group than the control group. Our results indicated that the SCL model gave full play to students’ subjective initiative, which was more effective and more satisfactory than the traditional teaching model. This can be explained by the following points. First, students’ active self-learning in preparation, including online literature browsing and previous knowledge, can fully activate students’ learning potential [25]. Second, students and teachers are actively involved in the whole teaching process, which changes the dominant position of teachers and makes students more active. These can better train students’ divergent and critical thinking abilities [26]. Third, the teacher selects the case that is closely related to the teaching content, and the case analysis process is that of understanding the professional theoretical knowledge, which can improve the students’ ability of using knowledge, analyzing, and solving problems [17].Our research results indicated that, in terms of reporting their scientific research thinking and comprehensive application ability, the benefit to the students in the SCL group was significantly better than that of the control group. This suggested that, compared with the traditional teaching model, the SCL model paid more attention to the training of students’ language organization and expression, and team cooperation ability, which can effectively improve students’ ability of independent thinking and analysis of problems.

The conclusions derived herein may be affected by the following factors. First, although we initially surveyed the math scores of college entrance examination and the scientific research work experience, students’ baseline status assessment is not comprehensive. Several factors could affect the results of students’ levels of satisfaction, such as the levels of statistics knowledge and the baseline levels of interest of expectation regarding statistics. Second, two teachers with the same professional title (both associate professors) were selected to participate in the teaching of statistics. To ensure the consistency of theoretical teaching between the two groups, the two teachers prepared and taught lessons uniformly according to the knowledge points of the teaching contents involved in the syllabus and adopted the same theory teaching model. Although there was no significant difference in the overall satisfaction of the theory course and teachers between two groups, the effect of the teacher on the results of this research is undeniable. Therefore, the research group will further refine and deeply collect baseline data to meet the principle of balance, so that the results are more convincing. At the same time, the subjective questions (application-oriented) and objective questions are further optimized and clarified to further verify that SCL method can improve students’ scientific research thinking and comprehensive application ability.

## Conclusions

In conclusion, compared with the traditional methods, as an effective method of high-quality education, the SCL model is worthy of further promotion in the practice teaching of preventive medicine.

## Data Availability

The data used during the study can be obtained from the corresponding author on reasonable request.

## Abbreviations

SCL: Seminar-case learning
LBL: Lecture-based learning
CBL: Case-based learning
χ^2^: Chi-square
KMO: Kaiser–Meyer–Olkin
K-S: Kolmogorov–Smirnov
